# Targeting Circadian Rhythm for the Regulation of Skin Collagen Metabolism

**DOI:** 10.1111/jocd.70638

**Published:** 2026-01-13

**Authors:** Cheng Wang, Tianlin Song, Yurong Zhang, Nihong Li, Ling Xie, Min Xie, Xingwu Jiang, Guanglei Lü, Yun Meng, Chaochao Wang, Lijun Yue, Wei Yang, Yang Li, Yelin Wu, Liang Chen

**Affiliations:** ^1^ Shanghai East Hospital, School of Medicine Tongji University Shanghai P. R. China; ^2^ Scientific Research Laboratory Shanghai Le‐Surely Biotechnology Co. Ltd Shanghai P. R. China; ^3^ SASELOMO Research Institute and Biological Laboratory Shanghai Chuanmei Industrial Co. Ltd Shanghai P. R. China; ^4^ Zhejiang Key Laboratory of Extreme Environment Functional Materials Yiwu Research Institute of Fudan University Yiwu China

**Keywords:** baicalin, circadian rhythm, collagen metabolism, palmitoyl tripeptide‐1, skin aging

## Abstract

**Background:**

Collagen is essential for maintaining skin structure and function, and the circadian rhythm is known to regulate a wide range of physiological processes.

**Aims:**

To investigate whether collagen metabolism in human skin fibroblasts exhibits circadian regulation, and to evaluate whether the time‐coordinated application of baicalin at daytime and palmitoyl tripeptide‐1 (PT‐1) at nighttime synergistically promotes collagen fiber formation and improves overall skin quality.

**Methods:**

A circadian‐synchronized human skin fibroblast model was established. The expression of collagen metabolism‐related genes was analyzed using qPCR and immunofluorescence. Subsequently, an 8‐week topical application study was conducted in mice using a regimen of daytime baicalin and nighttime PT‐1. Finally, a clinical trial involving 30 female participants was conducted, employing the same time‐coordinated application scheme.

**Results:**

Fibroblasts exhibited opposing day‐night rhythms: genes for collagen assembly (e.g., LOX) peaked during the day, while those involved in synthesis/secretion (e.g., Sec61a2, Mia3, Pde4d, Vps33b) and degradation (e.g., CTSK, MMP1) peaked at night. Time phase‐dependent interventions showed baicalin enhanced daytime assembly, while PT‐1 boosted nighttime synthesis. In mice, a timed day‐night combination therapy increased collagen fiber density. Clinical trial (*n* = 30) confirmed the efficacy, showing significant improvements in skin luminance (+16.29%), nasolabial fold depth (−36.35%), and firmness (R2: +24.35%).

**Conclusions:**

Collagen metabolism is regulated by circadian rhythms. Chronomodulated baicalin and PT‐1 application synergistically optimize collagen metabolism and improve skin quality.

## 1 Introduction

Collagen is the most abundant structural protein in the extracellular matrix of animal cells, accounting for approximately 20%–30% of total protein content in mammals [1, 2]. Collagen fibers are highly organized structures formed by the self‐assembly and covalent cross‐linking of collagen molecules, which confer structural integrity and elasticity to various tissues. In the skin, collagen makes up about 70% of dermal protein content and consists mainly of type I and type III collagen [3]. These types provide mechanical strength and elasticity, thereby maintaining the skin's structural stability and resistance to tensile stress. However, with aging, collagen synthesis declines and the structural organization of collagen fibers deteriorates, leading to visible signs of aging such as wrinkles and skin laxity. The biosynthesis of collagen and fiber formation involves a complex, multi‐step biological process including gene transcription, post‐translational modifications, secretion, transport, and fibrillar self‐assembly [4, 5, 6]. Therefore, a comprehensive understanding of the mechanisms underlying collagen synthesis and fiber assembly holds significant potential for developing strategies to delay skin aging.

Circadian rhythm is an endogenous timekeeping system that organisms have developed to adapt to the 24‐h light–dark cycle resulting from the Earth's rotation. It plays a crucial role in regulating a wide range of physiological processes, including the sleep–wake cycle, energy metabolism, endocrine function, and immune responses [7]. In recent years, increasing attention has been given to the regulatory influence of circadian rhythm on extracellular matrix proteins, particularly collagen metabolism. Research has demonstrated that core circadian clock components such as Mop3 and CLOCKΔ19 can indirectly modulate the expression of collagen genes in fibroblasts and influence collagen secretion and remodeling during tissue repair [8, 9]. Furthermore, experimental evidence suggests that disruptions in circadian rhythms may lead to an imbalance between the synthesis and degradation of extracellular matrix components, potentially accelerating tissue aging [10]. These studies mainly examine fibroblasts from the tendons and hearts of mice. However, whether collagen synthesis and fiber assembly in human skin fibroblasts are influenced by the circadian rhythm remains an area that requires further investigation. This research gap reveals an innovative way to boost fibroblast‐mediated fiber formation efficiency in the skin, leading to major progress in dermal regeneration and anti‐aging.

In recent years, a large number of small molecules or short peptides have been reported to have the function of increasing collagen. Baicalin, a flavonoid compound derived from scutellaria baicalensis, inhibits collagen degradation through the downregulation of matrix metalloproteinase expression, specifically MMP‐1 and MMP‐3 [11]. Vitamin C enhances type I collagen biosynthesis and stability by upregulating prolyl hydroxylase activity and downregulating matrix metalloproteinase expression [12]. Retinol and its derivatives activate the TGF‐β/Smad signaling pathway in fibroblasts, augment COL1A1 gene transcription, and facilitate collagen fibrillogenesis and assembly [13]. Moreover, novel collagen peptide‐based ingredients have been shown in both in vitro and in vivo studies to upregulate TGF‐β receptor expression and Smad signaling, thereby enhancing deposition of endogenous type I and III collagen fibers and improving skin elasticity and barrier function [14]. Palmitoyl tripeptide‐1 (PT‐1) is a synthetically derived palmitoylated oligopeptide that has been widely applied in the anti‐aging field through its ability to stimulate collagen synthesis [15, 16]. Niacinamide, as a precursor of NAD^+^, not only suppresses inflammation and oxidative stress by modulating PARP‐1 activity but also directly promotes gene expression and protein synthesis of type I, III, and V collagens in fibroblasts [17]. However, despite extensive mechanistic studies on how these molecules or peptides stimulate collagen synthesis, there remains a lack of systematic fundamental research addressing whether they regulate collagen biosynthesis and secretion in dermal fibroblasts via circadian rhythm–dependent pathways.

In this study, we elucidate the regulation of collagen metabolism—including its synthesis, secretion, assembly, and degradation in human skin fibroblasts, which has a circadian rhythm. Genes associated with collagen synthesis, secretion, and degradation are predominantly expressed during the nighttime phase, whereas genes involved in collagen assembly are primarily expressed during the daytime phase. Furthermore, PT‐1 enhances collagen synthesis during the night phase, while baicalin promotes fibril assembly during the day phase, thereby synergistically optimizing collagen metabolism. Both animal and human trials corroborate that these compounds significantly improve collagen fiber structure and skin function. This research innovatively reveals the circadian rhythm characteristics of collagen metabolism in human fibroblasts and identifies relevant regulatory molecules. Overall, this study provides novel perspectives for the development of collagen metabolism regulatory strategies based on the circadian rhythm.

## 2 Materials and Methods

### 2.1 Cell Culture

Human skin fibroblasts (HSFs) used in this study were purchased from Guangdong Biocell Biotechnology Co. Ltd., and were derived from the abdominal skin of a female donor. For cell isolation, fat and connective tissue were removed from the skin sample to improve cell purity. Subsequently, the tissue was digested overnight at 4°C with a 0.3% dispase (Sigma, Michigan, USA) solution to facilitate the separation of the epidermis and dermis. After separation, the dermis was cut into small pieces of approximately 1 mm^3^ and then digested with collagenase at 37°C for 2 h to break down the dermal collagen and release the fibroblasts. The resulting cell suspension was collected and seeded into T75 flasks. The culture medium consisted of low‐glucose DMEM (BasalMedia, Shanghai, China) supplemented with 10% FBS (Newzerum, New Zealand) and 1% penicillin–streptomycin (Beyotime, Shanghai, China). Cells were cultured in a humidified incubator at 37°C with 5% CO_2_. This method ensures the viability and proliferation capacity of HSFs, thus providing a consistent source of cells for subsequent experiments.

### 2.2 Construction of Cell Rhythm Models

HSFs were seeded at a density of 1 × 10^5^ cells/mL in 6‐well plates containing 2 mL of culture medium and incubated at 37°C for 24 h to allow cell attachment and growth. Subsequently, cells were treated with 100 nM dexamethasone (MCE, Shanghai, China) for 30 min to synchronize their state, followed by replacement with fresh medium and continued incubation for another 24 h; this time point was defined as zeitgeber time 0 (ZT0).

### 2.3 Real‐Time Quantitative PCR (qPCR)

HSFs subjected to different treatments at various time points were harvested and lysed using Trizol reagent for total RNA extraction. The extracted RNA was reverse‐transcribed into cDNA using the reverse transcription kit from Yeasen (11201ES08, Shanghai, China). According to the manufacturer's instructions, qPCR was performed using specific primers (listed in Table 1) and SYBR Green PCR reagents (11141ES60, Yeasen, Shanghai, China) to assess the expression levels of target genes.

**TABLE 1:** Oligonucleotides of primers.

Name of gene	Primers
Human GAPDH	F: CTTAGCACCCCTGGCCAAG R: TGGTCATGAGTCCTTCCACG
Human ATXN2L	F: GAACTAGCCGTGGATGCTGTG R: GCTGAATCGGTGAACTTGTCTT
Human CRY1	F: CTCCTCCAATGTGGGCATCAA R: CCACGAATCACAAACAGACGG
Human LOX	F: GCCGACCAAGATATTCCTGGG R: GCAGGTCATAGTGGCTAAACTC
Human CTSK	F: ACTCAAAGTACCCCTGTCTCAT R: CCACAGAGCTAAAAGCCCAAC
Human MMP1	F: GGGGCTTTGATGTACCCTAGC R: TGTCACACGCTTTTGGGGTTT
Human Sec61a2	F: ATTATGCAGTTGTTAGCTGGAGC R: CATACCAAACAGTTTCTGGGCT
Human Mia3	F: AAGTTCCAACAGATGAGACGGA R: GGTTCAGGTTCCCTTTCCTTAG
Human Pde4d	F: TGTGGCCTATCACAACAATATCC R: CACAGCCAAATGATGGTTCTCTA
Human Vps33b	F: TCAGACCCCGCATCAAGAATA R: ACCATCTCACACGCATAGAACT

### 2.4 Immunofluorescence Staining

HSFs subjected to different treatments at various time points were collected and fixed with 4% paraformaldehyde to preserve cellular morphology and structure. Cells were then permeabilized with 0.3% Triton X‐100 for 10 min to increase antibody accessibility. Non‐specific binding sites were blocked using 5% bovine serum albumin (BSA) for 30 min. The cells were incubated overnight at 4°C with anti‐PDI (455596S, Boston, USA) and anti‐COL1A1 (72026S, Boston, USA), followed by incubation with fluorescent secondary antibodies for 2 h at room temperature. Finally, cells were imaged using confocal microscopy to observe the expression and localization of target proteins.

### 2.5 Animal Experiments

Eight‐week‐old female C57BL/6 mice weighing approximately 20 g were obtained from Saiye Biotechnology Co. Ltd. (Suzhou, China). The mice were housed under controlled conditions (22°C ± 2°C, ~50%–60% humidity, 12 h light/12 h dark cycle) to maintain stable physiological status. All experimental procedures strictly complied with the National Standard for Laboratory Animal Welfare and Ethical Review of China (GB/T 35892‐2018) and were approved by the Animal Ethics Committee of the Shanghai East Hospital. During the study, mice had free access to food and water. Prior to treatment initiation, dorsal hair was removed. Baicalin (Zhejiang Moda Biotech Co. Ltd) was administered during the light phase (day time), while the PT‐1 (Shanghai Peptide Biotechnology Co. Ltd) was applied during the dark phase (night time). This regimen continued for 8 weeks. Following anesthesia with isoflurane, dorsal skin was harvested for subsequent analyses.

### 2.6 Tissue Transmission Electron Microscopy (TEM)

Mouse skin samples were fixed in electron microscopy fixative (Servicebio, Wuhan, China) at room temperature for 30 min to preserve cellular and tissue structure. Subsequently, the fixed skin was sectioned into approximately 1 mm^3^ pieces, which were immersed in fresh fixative and fixed overnight at 4°C. After fixation, samples were dehydrated, embedded, and imaged using TEM (HT7800, Hitachi, Japan) for collagen fiber analysis.

### 2.7 Tissue Staining and Collagen Imaging

Mouse skin tissues were first fixed in 4% paraformaldehyde at 4°C for 24 h. Following fixation, the tissues were rinsed with water for 30 min to remove residual fixative. The samples were then dehydrated with graded ethanol, cleared with xylene, and infiltrated with paraffin using a tissue processor (HistoCore PEARL, Leica, Germany). The tissues were embedded in paraffin and sectioned serially at 5‐μm thickness. Sections were deparaffinized in xylene and rehydrated through a graded ethanol series. Subsequently, the sections were stained with: Masson's trichrome for collagen visualization, Sirius Red for collagen subtype assessment, and hematoxylin and eosin (H&E) for general tissue morphology evaluation. For multiphoton imaging, a system based on a commercial laser scanning microscope (LSM 880, Zeiss, Germany) coupled to a femtosecond titanium: sapphire laser (Chameleon Super, Coherent, USA) was used, as described previously.

### 2.8 Clinical Evaluation

A prospective, randomized, single‐blind, controlled trial was conducted at ICAS Testing Technology Service (Shanghai) Co. Ltd., involving 30 Chinese Han women aged 27–40 years (mean ± SD: 37.67 ± 3.20 years) presenting with facial redness alongside visible signs of aging (e.g., skin sagging, wrinkles). Exclusion criteria included pregnancy, breastfeeding, planned pregnancy, history of cosmetic or serious allergies, systemic diseases, or severe skin conditions.

Participants applied a morning cream daily upon waking and a night cream nightly before bedtime for 8 weeks. Evaluations occurred at baseline, Week 2, Week 4, and Week 8. Prior to each assessment, subjects cleansed their faces, dried them with lint‐free paper, and acclimatized for 30 min in a controlled environment (20°C–22°C, 40%–60% humidity). Skin glossiness was measured using a Glossymeter GL200 (Courage+Khazaka). Mandibular angle contour and nasolabial fold severity were assessed using a PRIMOS‐CR roughness analyzer (LMI Technologies). Clinical photography and skin color (L* and a* values) were evaluated using the VISIA Complexion Analysis System (Canfield). Skin elasticity and firmness (R2 and F4 values) were evaluated using the Cutometer dual MPA580 (Courage+Khazaka). The day cream contained 0.15% Baicalin, while the night cream contained 3 × 10^−4^% PT‐1.

### 2.9 Statistical Analysis

Statistical analyses were performed using GraphPad Prism 8 (GraphPad Software, San Diego, CA, USA). Data are presented as mean ± SEM. Multiple group comparisons were analyzed by one‐way ANOVA followed by Tukey's *post hoc* test. For paired clinical data, normality was assessed; normally distributed data were analyzed using the paired t‐test, while non‐normally distributed data were analyzed using the Wilcoxon signed‐rank test. Statistical significance was set at *p* < 0.05; *p* ≥ 0.05 was considered not significant (ns).

## 3 Result

### 3.1 The Secretion, Assembly, and Degradation of Collagen in Human Skin Fibroblast Exhibit Circadian Rhythms

**FIGURE 1:**
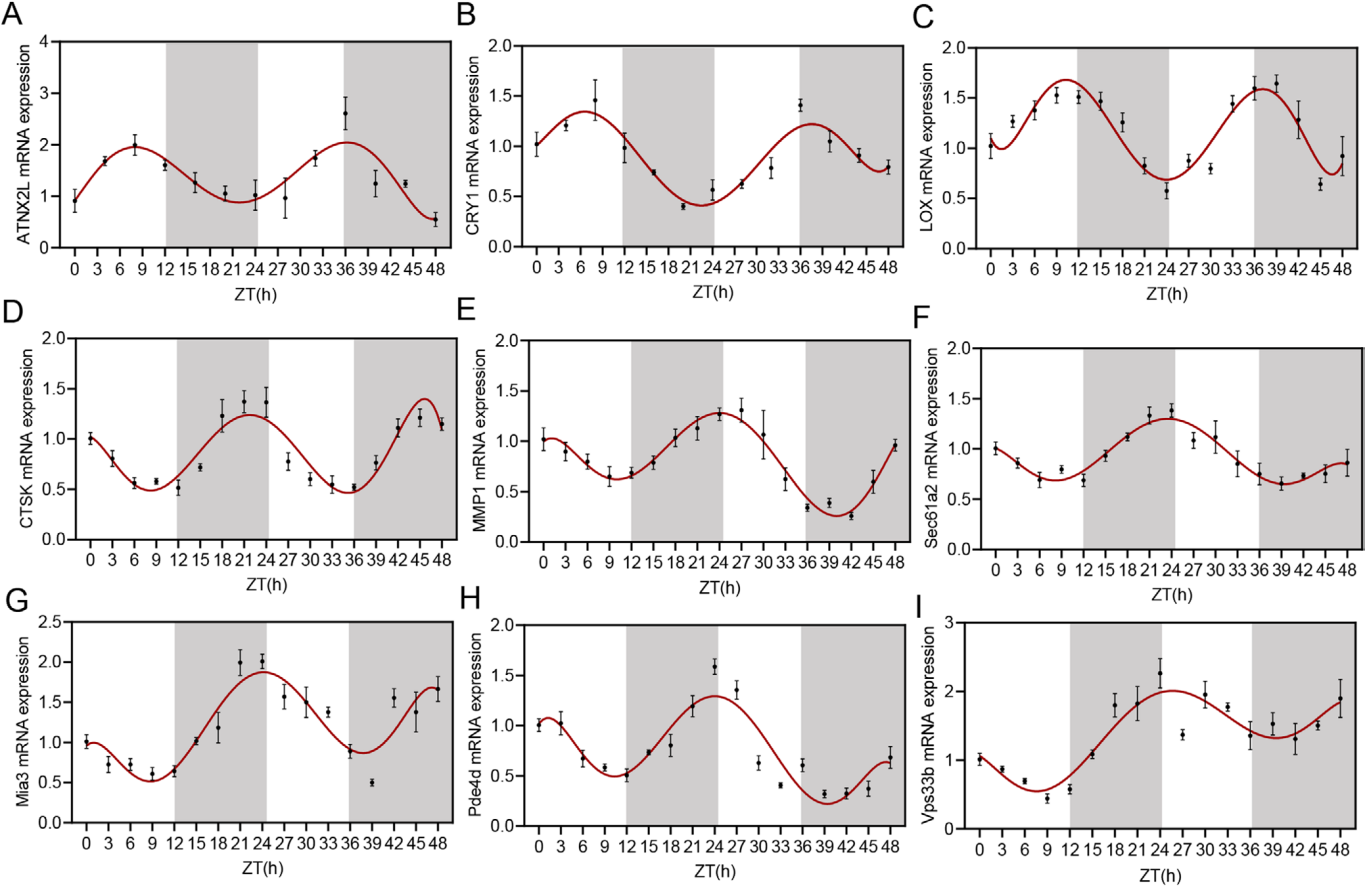
The collagen synthesis and assembly in HSFs show a circadian rhythm. qPCR measured the levels of rhythmic genes (A) ATXN2L and (B) CRY1. qPCR detected the expression of genes related to collagen fiber assembly LOX (C), genes related to collagen fiber degradation (D) CTSK, and (E) MMP1, and genes related to collagen synthesis and secretion (F) Sec61a2, (G) Mia3, (H) Pde4d, and (I) Vps33b. HSFs were seeded in 6‐well plates for 24 h. Subsequently, the cells were treated with 100 nM dexamethasone for 30 min. Total RNA was then extracted from the cells every 3 h post‐treatment (*n* = 3).

Human skin collagen, as the most abundant secretory protein in vertebrates, plays an important role in supporting skin structure and function. However, whether its secretion and assembly exhibit circadian rhythms has not been studied. Based on this, we constructed a circadian rhythm model of human skin fibroblasts (HSF), using dexamethasone to reset the expression of HSF biological genes to synchronize the rhythm. mRNA samples were collected every 4 h, and the expression levels of the rhythm genes ATXN2L and CRY1 were detected using qPCR. Curve fitting analysis of the qPCR revealed that within 48 h, the mRNA levels of ATXN2L and CRY1 exhibited a periodic trend of two approximate sine waves, indicating that the rhythm model of HSF cells was successfully prepared (Figure 1A,B). Meanwhile, we found that key enzymes related to collagen cross‐linking and tissue remodeling, such as lysyl oxidase (LOX), the main degradation enzyme for collagen fibers, cathepsin K (CTSK), and matrix metalloproteinase 1 (MMP1), as well as proteins related to collagen synthesis and secretion, including Sec61a2, Mia3, Pde4d, and Vps33b, all exhibited a circadian rhythm. The mRNA expression of LOX, associated with collagen fiber assembly, rose during the day phase and fell during the night phase; on the other hand, the mRNA expression of genes related to collagen synthesis and secretion (Sec61a2, Mia3, Pde4d, and Vps33b) and the mRNA expression of CTSK and MMP1, related to collagen degradation, gradually decreased during the day phase and gradually increased during the night phase (Figure 1C–I). These rhythmic expressions indicate that the biological processes related to collagen synthesis, secretion, and degradation mainly occur during the night phase, while processes related to collagen assembly mainly occur during the day phase.

**FIGURE 2:**
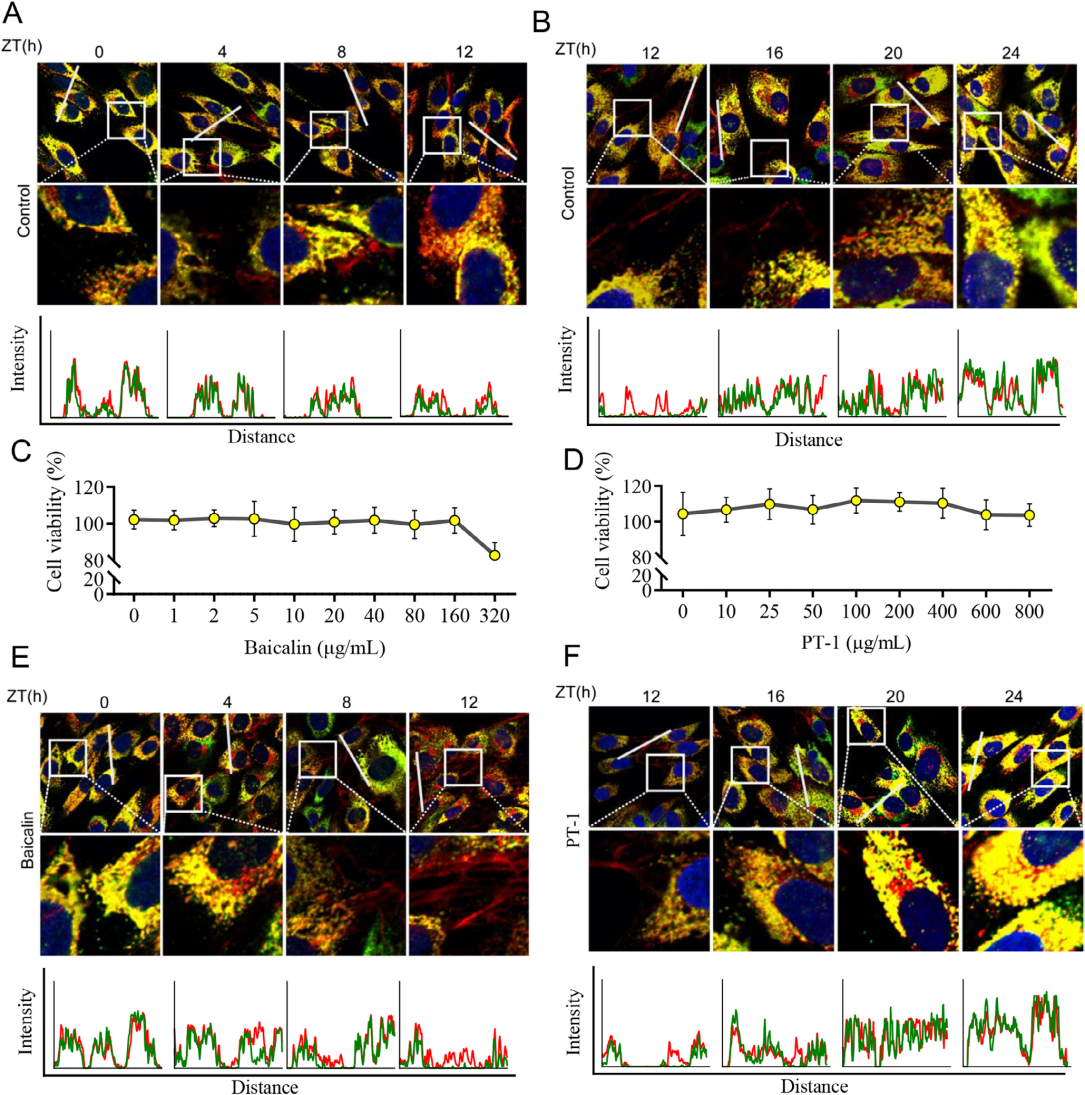
Baicalin enhances collagen assembly, while PT‐1 stimulates collagen synthesis. (A, B) Assessment of the rhythmic patterns of collagen synthesis and assembly through immunofluorescence. Rhythmic model cells were collected at various culture time points to evaluate the expression levels of PDI (green) and Collagen I (red) via immunofluorescence staining. (C, D) Determination of the safe concentration ranges for baicalin and PT‐1. HSF were cultured in 96‐well plates for 24 h, followed by treatment with varying concentrations of baicalin and PT‐1. Cell viability was assessed using the CCK‐8 assay to determine the optimal non‐toxic concentrations. (E, F) Immunofluorescence analysis demonstrating the effects of baicalin on collagen assembly and PT‐1 on collagen synthesis. Rhythmic model cells treated with baicalin and PT‐1 were collected at various culture time points to evaluate the expression levels of PDI (green) and Collagen I (red) via immunofluorescence staining.

To further demonstrate that the processes of collagen synthesis, secretion, degradation, and assembly are regulated by circadian rhythms, we used confocal microscopy to observe the synthesis and assembly of collagen regulated by circadian rhythms. Type I procollagen (Collagen I) is first synthesized in the endoplasmic reticulum and then secreted into the extracellular matrix, where it matures into collagen after being cleaved by proteases. Mature collagen can self‐assemble into highly ordered collagen fibers. Therefore, if Collagen I protein co‐localizes with protein disulfide isomerases (PDI) related to the oxidative folding of nascent peptide chains in the endoplasmic reticulum (shown in yellow in the images), this means collagen is present. Conversely, if Collagen I protein does not co‐localize with PDI (shown in red in the images), it indicates the presence of collagen fibers. As shown in the staining results of the rhythmic model cells, red fibrous collagen is mainly produced during the day phase, while yellow collagen is mainly produced during the night phase (Figure 2A,B).

### 3.2 Baicalin Promotes Collagen Assembly While PT‐1 Can Promote Collagen Synthesis

It has been reported that complexes containing PT‐1 can significantly promote fibroblast collagen synthesis [18], while baicalin can inhibit the expression of matrix metalloproteinases [19]. We then examined whether PT‐1 and baicalin affect collagen secretion and assembly in a circadian rhythm. First, through cell viability assays, we confirmed that baicalin at a concentration of 160 μg/mL and PT‐1 at 800 μg/mL showed no significant cytotoxicity to HSF, making sure the following experiments were safe and effective (Figure 2C,D). Subsequently, we treated HSF cells with baicalin under day phase culture conditions and treated the cells with PT‐1 under night phase conditions. Using immunofluorescence co‐staining techniques for PDI and Collagen I, we dynamically observed the synthesis and secretion of collagen and the assembly of collagen fibers. The results showed that baicalin treatment significantly promoted the assembly of collagen fibers, with filamentous collagen fibers further increasing in the extracellular matrix, while PT‐1 treatment significantly enhanced the synthesis level of Collagen I, with increased co‐localization signals of PDI and Collagen I in the endoplasmic reticulum, showing an active collagen synthesis process (Figure 2E,F). This result indicates that PT‐1 and baicalin play synergistic roles in promoting synthesis and assembly during the night and day phases, respectively.

### 3.3 The Day‐Night Combinational Use of Baicalin and PT‐1 Promotes Collagen Fiber Formation in the Mouse Skin

**FIGURE 3:**
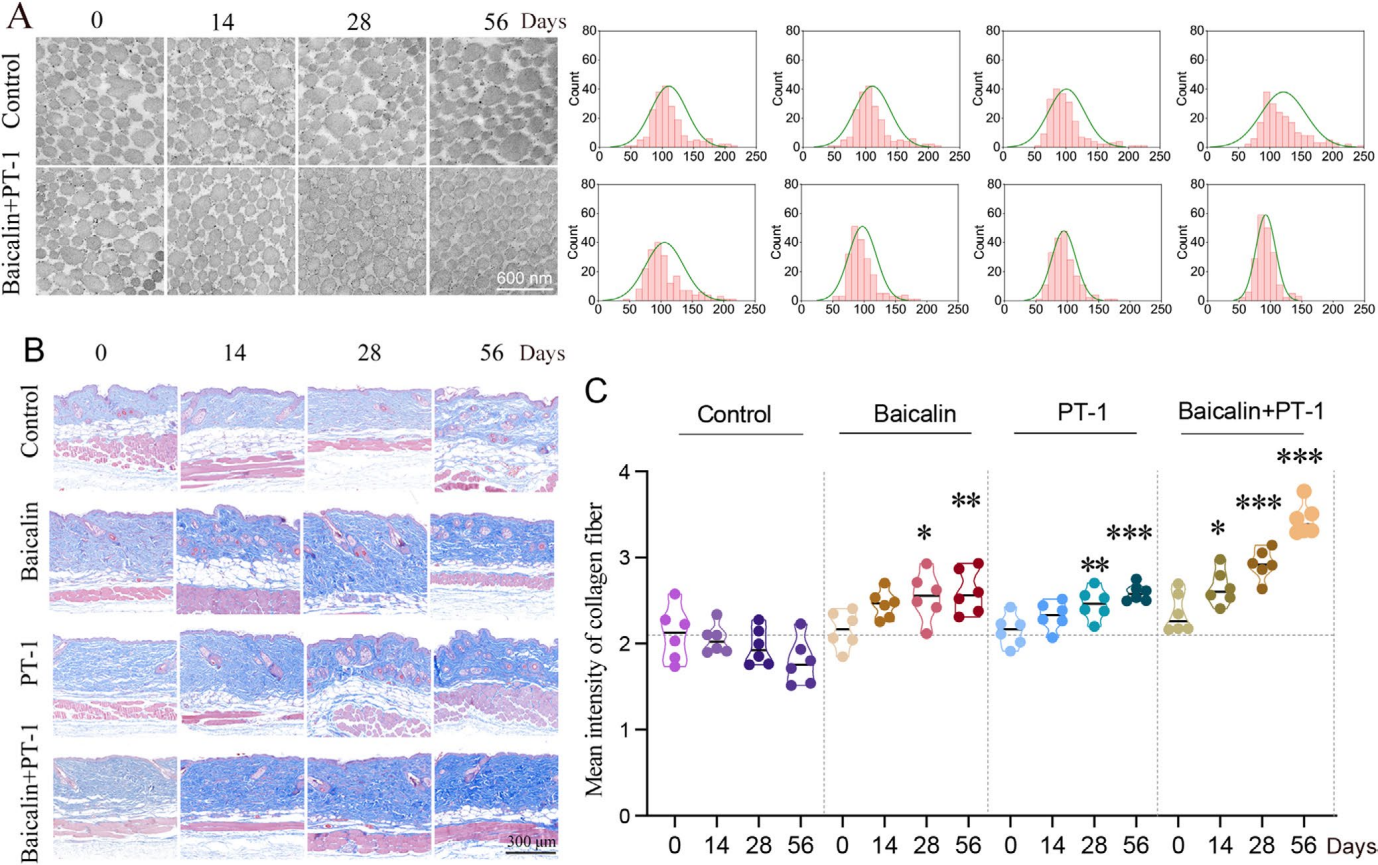
The combined application of baicalin during daytime and PT‐1 nighttime enhances collagen fiber formation in mice. The dorsal skin of the mice was treated with baicalin during the day and with PT‐1 at night. Skin samples were collected at 0, 4, 28, and 56 days post‐treatment for evaluation. (A) TEM was employed to assess fiber structure in the dorsal skin of mice. (B) Collagen fibers were evaluated using Masson's trichrome staining. (C) Collagen fibers were quantified (*n* = 6. *p < 0.05, **p < 0.01, ***p < 0.001).

**FIGURE 4:**
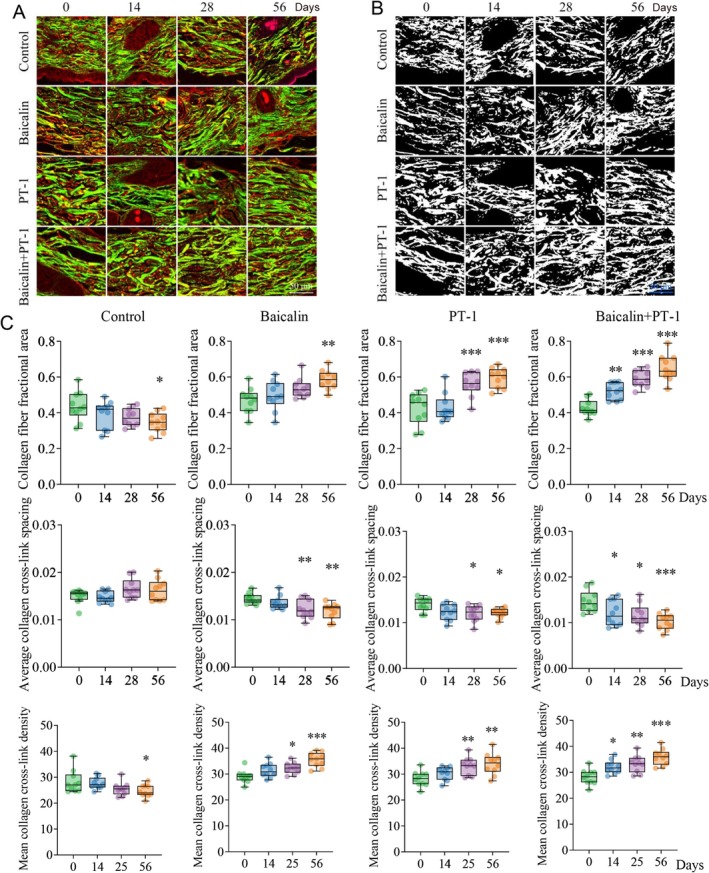
Two‐photon microscopy reveals that daytime application of baicalin and nighttime application of PT‐1 promotes collagen fiber formation in mice. The dorsal skin of the mice was treated with baicalin during the day and with PT‐1 at night. Skin samples were collected at 0, 4, 28, and 56 days post‐treatment for evaluation. (A, B) Two‐photon microscopy was utilized to examine skin fiber. (C) Analysis of collagen fiber fractional area, mean collagen cross‐link spacing and average collagen cross‐link density. (*n* = 10. *p < 0.05, **p < 0.01, ***p < 0.001).

Based on the above, we checked whether Day‐night combinational use of baicalin and PT‐1 could help collagen fiber formation in mice. We treated the dorsal skin of mice with PT‐1 and baicalin during the night and day phases, respectively, for 56 days. Afterward, we assessed the expression of collagen using techniques such as TEM, collagen staining, and two‐photon microscopy. As shown, TEM images indicated that the diameter of collagen fibers tended to be uniform, with a more orderly and compact arrangement, and the gaps between fibers significantly reduced, indicating effective improvement in collagen fiber synthesis and assembly. Measurements of collagen fiber diameter also indicated that the diameter of collagen fibers tended to be uniform and slender (Figure 3A) after treatment with PT‐1 and baicalin. Masson staining analysis also showed a significant increase in collagen fiber content in the skin of mice after treatment with PT‐1 and baicalin (Figure 3B,C). Additionally, we used two‐photon microscopy to detect collagen fibers after treatment with PT‐1 and baicalin. As shown in Figure 4, the collagen fiber area and average cross‐link density increased significantly, while the mean cross‐link spacing decreased, indicating a tighter collagen arrangement. In summary, the Day‐night combinational use of baicalin and PT‐1 effectively improves the state of skin fibers by promoting collagen synthesis and the orderly assembly of collagen fibers.

### 3.4 The Day‐Night Combinational Use of Baicalin and PT‐1 Promotes Collagen Formation in Human Skin

**FIGURE 5:**
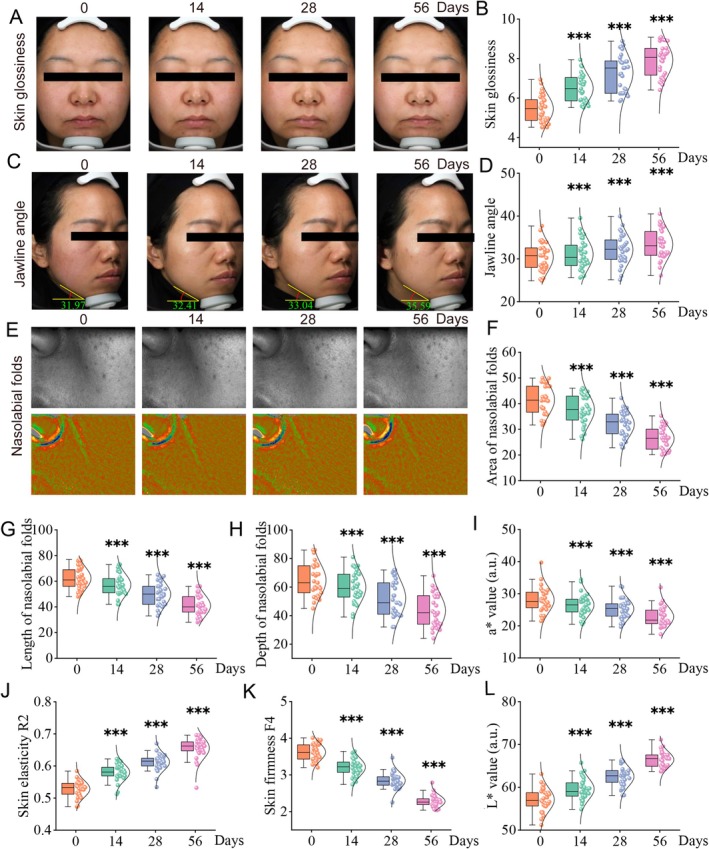
The combined application of baicalin and PT‐1 during daytime and nighttime enhances human collagen production. (A, B) Assessment of skin glossiness and its quantitative measurement. (C, D) Evaluation of mandibular angle contour and corresponding quantification. (E–H) Analysis of nasolabial fold length, depth, and surface area. (I) Measurement of skin redness (a* value) and its quantification. (J) Skin elasticity represented by the R2 value. (K) Skin firmness indicated by the F4 value. (L) Skin color assessed by the L* value (*n* = 30. *p < 0.05, **p < 0.01, ***p < 0.001).

To further explore the effects of the Day‐night combinational use of PT‐1 and baicalin on human skin collagen fibers, we conducted an 8‐week test on 30 subjects, all of whom did not experience local erythema, swelling, edema, or systemic adverse reactions. The study results found that the combined use of PT‐1 and baicalin effectively improved skin luminosity and enhanced the jawline (Figure 5A–D). Primos‐Lite data showed that the nasolabial folds were significantly improved, with a reduction of 23.05% and 36.35% at 28 and 56 days, respectively after the treatment (Figure 5E–H). Meanwhile, the a* value, which indicates skin redness, significantly decreased by 10.17% on day 28 and by 18.83% on day 56 (Figure 5I). Cutometer test results showed a significant increase in skin firmness, with the R2 value improving by 16.10% after 28 days and further increasing to 24.35% after 56 days; F4 value decreased by 21.31% on day 28 and continued to decrease to 36.5% by day 56, with statistically significant differences compared to baseline, indicating enhanced skin elasticity (Figure 5J,K). Additionally, the L* value, reflecting skin pigmentation, significantly increased by 9.28% after 28 days and reached an increase of 16.29% by day 56 (Figure 5L). In conclusion, the combined use of baicalin and PT‐1 can boost skin brightness, enhance the jawline and skin firmness, improve nasolabial folds, and ultimately promote skin health by promoting collagen fiber formation.

## 4 Discussion

The synthesis and degradation of collagen play a critical role in maintaining the structural integrity and functional properties of the skin. This study systematically investigated the circadian rhythm‐regulated mechanisms underlying collagen metabolism using a dexamethasone‐synchronized human skin fibroblast model. The findings revealed that the processes of collagen synthesis, secretion, and assembly are tightly modulated by the circadian clock. Furthermore, PT‐1 and baicalin were found to significantly influence collagen synthesis and assembly during daytime and nighttime phases, respectively. These results not only elucidate the rhythmic patterns in collagen synthesis and assembly but also offer a novel framework for understanding and regulating skin collagen fiber dynamics.

This study suggests that genes associated with collagen synthesis in human fibroblasts, including Sec61a2, Mia3, Pde4d, and Vps33b, as well as collagen‐degrading enzymes such as CTSK and MMP1, exhibit increased expression during the nighttime phase and reduced expression during the daytime phase. In contrast, LOX, an enzyme involved in collagen assembly, demonstrates elevated expression during the daytime and decreased expression at night. These findings align with those reported by Chang et al., who observed circadian patterns in collagen synthesis and assembly within mouse tail tendons [20]. Furthermore, Yao's research confirms that disruption of the circadian rhythm can lead to an imbalance in collagen homeostasis, with the core circadian clock gene BMAL1 regulating collagen expression via the Sirtuin‐1 pathway. This provides a mechanistic basis supporting the hypothesis of this study that collagen fiber synthesis and assembly in humans follow a circadian rhythm [21]. Additionally, in studies on pulmonary fibrosis, Wang et al. found that clock protein REV‐ERBα significantly suppresses the expression of COL1 and lysyl oxidase, thereby modulating fibrotic homeostasis [22]. Moreover, fibroblasts deficient in the clock protein NPAS2 display abnormal proliferation and collagen fiber remodeling [23], further indicating that the circadian rhythm can directly influence collagen homeostasis. Collectively, these studies substantiate the hypothesis of this study that collagen fiber synthesis and degradation exhibit circadian rhythmicity.

With advancing age in mice, dermal collagen fibril diameter progressively increases, fibril organization becomes disordered, and interfibrillar spacing markedly widens, indicating that collagen fibril structural degeneration is closely linked to skin aging. Previous studies have shown that the COL‐I/COL‐III ratio rises with age, from 1.3:1 at week 0 to 4.5:1 by week 9, and that collagen fibril diameter increases from 40 to 112 nm [24]. Atomic force microscopy further confirmed that aged skin exhibits rougher collagen fibril surfaces and increased fibril bundle stiffness, correlating with elevated MMP‐1 expression and accumulation of advanced glycation end‐products (AGEs) [25]. Upon treatment with PT‐1 and baicalin, collagen fibril diameters converge toward uniformity, fibrils align in a more ordered and compact fashion, and interfibrillar gaps are substantially reduced, underscoring the importance of fibril structural stability for skin integrity. These morphological alterations directly reflect changes in skin mechanical strength and elasticity. Our study found that combined PT‐1 and baicalin treatment results in homogeneous fibril diameter, tighter and more regular fibril packing, and decreased interfibrillar spacing, highlighting the critical role of collagen fibril stability in skin health and elasticity. An eight‐week clinical trial also corroborated that their combined use safely and effectively enhances skin brightness and elasticity while reducing wrinkles and transepidermal water loss, in agreement with multiple clinical investigations of collagen‐supplementation products [26, 27, 28]. These findings provide clinical justification for their incorporation into dermocosmetic formulations.

We further confirmed that the small molecule baicalin and the polypeptide PT‐1 effectively enhance the synthesis and assembly of collagen. Moreover, the combined application of baicalin and PT‐1 during daytime and nighttime enhances human collagen production. The synergistic efficacy of timed application, validated in mice and humans, establishes “chronocosmeceuticals” as a novel precision anti‐aging strategy. Unlike conventional continuous dosing, this interdependent approach minimizes metabolic conflicts, such as simultaneously stimulating collagen fiber synthesis and degradation, and enhances bioavailability. However, the mechanisms by which baicalin and PT‐1 regulate circadian genes to promote collagen synthesis and assembly remain to be further investigated.

In our experimental design, mice were raising under standard laboratory light–dark cycles consisting of 12‐h light and 12‐h dark periods. the circadian phase of animals is primarily entrained by photic cues rather than by endogenous behavioral activity [29, 30]. Although mice are nocturnal, the artificial light phase is interpreted by the suprachiasmatic nucleus (the central pacemaker) as their subjective day. This central timing is subsequently communicated to synchronize peripheral oscillators in tissues like the skin [31]. Consequently, during the laboratory day, mouse skin enters a phase dominated by collagen assembly, while during the laboratory night, it shifts to a phase favoring collagen synthesis. This phasing is comparable to the human skin circadian rhythm, which is also entrained by the light–dark cycle. Therefore, our dosing regimen remains physiologically consistent with the intended human application and does not require inversion.

This study systematically reveals for the first time the circadian characteristics of collagen biosynthesis and fibril assembly, as well as the rhythmic expression of their key regulatory genes, demonstrating that collagen synthesis and collagen fibrillogenesis are two relatively autonomous processes governed by alternating day–night control. We show that baicalin and PT‐1 selectively enhance collagen assembly and collagen synthesis, respectively, in a circadian context, synergistically optimizing collagen metabolism and markedly improving skin architecture and function. These findings provide novel targets and a theoretical framework for rhythm‐based anti‐aging therapeutic strategies. This research also provides a new direction for collagen‐based tissue regeneration and repair strategies.

## Author Contributions

Cheng Wang: conceptualization, formal analysis, investigation, methodology, visualization, writing – original draft. Tianlin Song: formal analysis, investigation, writing – original draft. Yurong Zhang: formal analysis, investigation, funding acquisition, writing – original draft. Nihong Li: formal analysis, investigation, resources. Ling Xie: formal analysis, investigation, resources. Min Xie: formal analysis, investigation, resources. Xingwu Jiang: formal analysis, investigation. Guanglei Lü: formal analysis, investigation. Yun Meng: formal analysis, investigation. Chaochao Wang: formal analysis, investigation. Lijun Yue: formal analysis, investigation. Wei Yang: supervision, resources. Yang Li: conceptualization, methodology, project administration, supervision, visualization, resources, writing – review and editing. Yelin Wu: conceptualization, funding acquisition, methodology, project administration, supervision, visualization, writing – review and editing. Liang Chen: conceptualization, methodology, project administration, supervision, visualization, resources, writing – review and editing.

## Funding

This work was supported by the National Natural Science Foundation of China (Grants 82172091, 82402450, 82372122 and U25A20245), the Shanghai Science and Technology Innovation Action Plan (Grants 23XD1422800 and 23S31900200), and The Medical Discipline Construction Program of Shanghai Pudong New Area Health Commission (the Emerging and Interdisciplinary Disciplines Program, PWXx2025‐03).

## Ethics Statement

The study was approved by the Shanghai Ethics Committee for Clinical Research (SECCR2025‐173‐01) and followed the Declaration of Helsinki. Benefits, risks, and potential complications were explained to the subjects. All subjects voluntarily participated in this study and signed an informed consent form.

## Conflicts of Interest

The authors declare no conflicts of interest.

## Data Availability

The main data supporting the results in this study are available within the paper and its Supporting Information Data available on request from the authors. The data that support the findings of this study are available from the corresponding author upon reasonable request.
